# Association between ambient PM_2.5_ and outpatient visits of children's respiratory diseases in a megacity in Central China

**DOI:** 10.3389/fpubh.2022.952662

**Published:** 2022-09-30

**Authors:** Le Liu, Bingya Wang, Nana Qian, Huiyan Wei, Guangmei Yang, Leping Wan, Yan He

**Affiliations:** ^1^Department of Environment Health, School of Public Health, Zhengzhou University, Zhengzhou, China; ^2^Department of Nutrition, People's Hospital of Zhengzhou, Zhengzhou, China; ^3^Department of Radiology, The Second Affiliated Hospital of Zhengzhou University, Zhengzhou, China; ^4^Department of Social Medicine and Health Administration, School of Public Health, Zhengzhou University, Zhengzhou, China

**Keywords:** PM_2.5_, children, respiratory disease, outpatient visits, generalized additive model

## Abstract

**Objective:**

To explore the relationship between ambient PM_2.5_ level and outpatient visits of children with respiratory diseases in a megacity, Zhengzhou, in central China.

**Methods:**

We collected daily outpatient visit data, air pollutant data, and meteorological data at the monitoring points of Zhengzhou from the time period 2018 to 2020 and used Spearman's rank correlation to analyze the correlation between children's respiratory outpatient visits and air pollutants and meteorological factors. Generalized additive models were used to analyze the association between PM_2.5_ exposures and children's respiratory outpatient visits. A stratified analysis was further carried out for the seasons.

**Results:**

From 2018 to 2020, the total number of outpatients with children's respiratory diseases was 79,1107, and the annual average concentrations of PM_2.5_, PM_10_, SO_2_, NO_2_, CO, and O_3_-8h in Zhengzhou were respectively 59.48 μg/m^3^, 111.12 μg/m^3^, 11.10 μg/m^3^, 47.77 μg/m^3^, 0.90 mg/m^3^ and 108.81 μg/m^3^. The single-pollutant model showed that the risk of outpatient visits for children with respiratory disease increased by 0.341% (95%*CI*: 0.274–0.407%), 0.532% (95%*CI*: 0.455–0.609%) and 0.233% (95%*CI*: 0.177–0.289%) for every 10 μg/m^3^ increase in PM_2.5_ with a 3-day lag, 1-day lag, and 1-day lag respectively for the whole year, heating period, and non-heating period. The multi-pollutant model showed that the risk of PM_2.5_ on children's respiratory disease visits was robust. The excess risk of PM_2.5_ on children's respiratory disease visits increased by 0.220% (95%*CI*: 0.147–0.294%) when SO_2_ was adjusted. However, the PM_2.5_ effects were stronger during the heating period than during the non-heating period.

**Conclusion:**

The short-term exposure to PM_2.5_ was significantly associated with outpatient visits for children's respiratory diseases. It is therefore necessary to strengthen the control of air pollution so as to protect children's health.

## Introduction

Mounting evidence shows a clear association between the incidence of respiratory diseases and the concentration of air pollutants (1, 2). Air pollution has become an urgent public health problem in developing countries. Previous studies have shown that children are more susceptible to air pollution because of their higher breath rate, undeveloped lungs, and longer time playing outdoor (3–5). Air pollution has been demonstrated to be the most common cause of death and morbidity due to respiratory diseases in children all over the world (6, 7). However, the impact of air pollutants on children's respiratory diseases should be different in cities with different levels and sources of air pollution and meteorological factors.

PM_2.5_, as a major pollutant affecting air quality, easily becomes the carrier of toxic substances in the air, thereby having a great impact on human health (8–10). PM_2.5_ can be inhaled into the lungs and cause respiratory diseases such as asthma, pneumonia, and upper respiratory tract infections. The spread and transformation of particulate matter will also affect the atmospheric visibility and global climate, etc. (11, 12), especially in northern China, where there is a high level of air pollution (13).

Zhengzhou, located in 112°42'- 114°14' E, 34°16'- 34°58'N, known as the political, economic, and cultural center of Henan Province is a megacity with a large population base in central China. It is not only an important industrial city, but also an important transportation hub that connects China's north-south and east-west major railway and expressway lines, forming an intersection of China's transportation systems. Additionally, Zhengzhou has a warm temperate continental climate with four distinct seasons. It is cold in the winter and the daily average temperature is usually lower than 5°C, and so, central heating in buildings operates throughout the whole winter. The air pollution in winter is usually higher than other seasons due to heating.

The purpose of our study is to explore the short-term effect of PM_2.5_ on the outpatient visits of children with respiratory diseases and the outpatient volume during the heating period in Zhengzhou City and further provide a basis for air pollution prevention, reducing the risk of children's respiratory diseases and formulating relevant health policies.

## Materials and methods

### Date acquisition

#### Data on daily outpatient visits

The daily outpatient data of children's respiratory diseases in Zhengzhou from 2018 to 2020 were obtained from two representative large children's hospitals (Henan Provincial Maternal and Child Health Hospital and Henan Provincial Children's Hospital), which both used the electronic medical record system. The registration contents consisted of outpatient visit date, gender, birth date, age, contact address, preliminary diagnosis, International Classification of Disease, 10th Revision (ICD-10) codes, and department. The information of all patients was exported by the staff of the hospital information center, and then the children aged 0–14 years living in Zhengzhou were selected as study cases. These cases were coded according to the ICD-10 codes for respiratory diseases (J00–J99).

#### Air pollution data

Daily air pollution data from January 1, 2018 to December 31, 2020 were provided by Zhengzhou Environmental Protection Monitoring Center. The daily mean values of 9 fixed national monitoring points in the urban district of Zhengzhou were used as the daily mean concentrations of pollutants in the atmosphere. These data included the daily average concentration of fine particles (≤2.5 μm, PM_2.5_), inhalable particles (≤10 μm, PM_10_), sulfur dioxide (SO_2_), nitrogen dioxide (NO_2_), carbon monoxide (CO) and the maximum 8-h average daily concentration of ozone (O_3_-8h).

#### Meteorological data

The meteorological data of the same period were collected by Zhengzhou Meteorological Bureau, including daily average air temperature, daily average pressure, daily average relative humidity, daily average wind speed, and hazy days.

### Data processing and statistical analyses

The annual missing rates of air pollutant data and meteorological data were both < 5%. The missing values of air pollutants in each national controlled air quality monitoring station were calculated by using the same pollutant data from other monitoring stations at the same time according to the linear regression interpolation method. The same method was also used to fill in missing parts of meteorological data, and a logical check was then performed to ensure the data are reasonable.

SPSS 21.0 software was used for description analysis and correlation analysis. The number of outpatient visits for children with respiratory system diseases in Zhengzhou City, the concentration of air pollutants (PM_2.5_, PM_10_, SO_2_, NO_2_, CO, O_3_-8h), and meteorological indicators were descriptively analyzed, including mean ± standard deviation (*Mean* ± *SD*), minimum (*Min*), median (*M*), maximum (*Max*), 25th percentile (P_25_) and 75th percentile (P_75_).

The Kolmogorov-Smirnov test was used to examine whether the data fit normal distribution. Results suggested that the outpatient visit data did not conform to the normal distribution (*P* < 0.001), so Spearman's correlation coefficients were used to evaluate the inter-relations among air pollutant concentration, meteorological parameters, and the number of outpatient visits for children with respiratory diseases. *P-*values < 0.05 were considered to indicate statistical significance.

R3.6.2 software was used to establish the time series analysis model. Compared with the total population of Zhengzhou, the risk of daily visits to children for respiratory diseases represent a small probability event and follow a Poisson distribution (14). Time series Poisson generalized additive model (GAM) was used to estimate the associations between daily mean concentration of air pollutant and daily visits for children with respiratory diseases, and adjust the time trend, day of the week effect, meteorological parameters, and other confounding factors. The natural spline smoothing function was used to control for long-term trend, daily mean temperature, relative humidity, and average pressure effect. Indicator variables were used to control the effects of weekdays and holiday effect (15), as well as seasonal patterns. According to Akaike's Information Criterion (AIC), an appropriate degree of freedom was specified. The smaller the AIC value, the better the model fit (16–18). The degree of freedom of the final determination time was 7 per year, and each meteorological index takes 3 per year (19, 20). The model was described below:


$$\begin{array}{l} Log\left[E\left(Y_{i}\right)\right]=\beta X_{i}+ns\left(Time,df\right)+DOW+ns\left(Z_{i,}df\right)+\;intercept \end{array}$$

where *i* refers to the day of the observation, E(Yi) represents the expected number of children's respiratory disease outpatients on day *i*; *X*_*i*_ represents the pollutant concentrations on day *i*; β is the exposure-response relationship coefficient, that is, the increase in the number of visits for children with respiratory diseases caused by the increase in the concentration of pollutants per unit; *ns* indicates the natural cubic regression smooth function; time is the data variable, *df* represents the degrees of freedom. *DOW* indicates the dummy variable for the day of the week on day *i*; *Z*_*i*_ is the meteorological factor on day *i*, including average daily temperature, average daily relative humidity, wind speed, and air pressure.

Both single-pollutant and multi-pollutant models were fitted with different combinations of pollutants to assess the stability of the impact of major air pollutants on outpatient visits for children with respiratory disease. Delay effect was considered to investigate the single-day lags (from lag0 to lag7) for air pollutants. A lag of 0 day (lag0) refers to the current-day air pollutant concentration and a lag of 1 day (lag1) refers to the previous-day's air pollutant concentration. Calculate the percentage increase or decrease in the risk of daily outpatient visits for children with respiratory diseases (Excess risk, *ER*) and 95% confidence interval (95%*CI*) for every 10μg/m^3^ increase in the PM_2.5_ concentration.

To establish the exposure-response relationship between air pollutants and the increasing percentage of children's respiratory diseases, the pollutant concentration of the maximum health effect days was taken as the exposure level. The pollutants were introduced into the model as a spline smoothing function (degree of freedom was set to 3) to understand the relationship between pollutant concentration and health effects.

Then other air pollutants were introduced at the same time for multi-pollutant model fitting, and collinearity was controlled by a stepwise regression method. The concentration of each air pollutant entered the model in turn, and different lag days were selected (heating and non-heating).

## Results

### Descriptive analysis

During 2018–2020, the total number of outpatient visits for children with respiratory diseases was 791,107 with the daily mean visits of 1,079, the maximum daily visits of 3,100, and the minimum daily visits of 322. The daily mean concentrations of SO_2_ and CO were respectively 11.10 μg/m^3^ and 0.90 mg/m^3^, which was below the national level 1 concentration limits (SO_2_:50 μg/m^3^; CO:4 mg/m^3^) according to Ambient Air Quality Standard'(AQS) (21). The concentration of O_3_-8h was below the AQS level 2 concentration limit (160 μg/m^3^). The mean concentrations of PM_2.5_, PM_10_, and NO_2_, were 59.48 μg/m^3^, 111.12 μg/m^3^, and 47.77 μg/m^3^, which exceeded the Level 2 concentration limits of the AQS (35 μg/m^3^,70 μg/m^3^ and 40 μg/m^3^) and also exceeded the World Health Organization (WHO) air quality guidelines (AQG), as shown in Table 1.

**Table 1:** Descriptive analysis of air pollutant concentrations, meteorological parameters, and outpatient visits.

**Variables**	***Mean ± SD***	**Min**	***P*_25_**	***M***	***P*_75_**	**Max**
**Air pollutants**						
PM_2.5_(μg/m^3^)	59.48 ± 45.59	7.0	28.0	41.0	71.0	334.0
PM_10_(μg/m^3^)	111.12 ± 53.98	16.0	73.0	99.0	138.4	308.5
SO_2_(μg/m^3^)	11.10 ± 6.64	1	6.0	9.5	13.0	46.0
NO_2_(μg/m^3^)	47.77 ± 20.52	10.22	33.0	45.5	57.5	126.0
CO(mg/m3)	0.90 ± 0.36	3.0	0.7	0.8	1.1	2.5
O_3_-8h(μg/m3)	108.81 ± 57.59	7.0	59.5	102.5	157.8	247.5
**Meteorological parameters**						
Temperature (°C)	16.57 ± 10.25	−3.0	7.2	17.2	26.2	33.6
Pressure (kPa)	1003.61 ± 9.86	984.7	1026.7	994.7	1003.5	1010.8
Relative humidity (%)	55.70 ± 17.24	18.0	43.0	56.0	69.0	93.0
**Outpatient visits**						
Total	1078.82 ± 627.95	322.0	597.5	864.0	1392.8	3100.0
Heating period	1196.99 ± 660.81	350.0	653.0	924.0	1524.0	3100.0
Non–heating period	962.59 ± 571.34	322.0	495.3	845.0	1129.8	2720.0

### Correlation analysis

Kolmogorov-Smirnov test results showed that the air pollutants and meteorological factors do not conform to the normal distribution (*P* < 0.05). Spearman's correlation analysis showed that PM_2.5_ was positively correlated with PM_10_, SO_2_, NO_2_, CO, relative humidity, and air pressure (*r* > 0, *P* < 0.05), and negatively correlated with O_3_-8h and average daily temperature (*r* < 0, *P* < 0.05). There were significant correlations between the 6 air pollutants (*P* < 0.01). O_3_-8h was negatively correlated with the other five pollutants, air pressure, and relative humidity (*r* < 0, *P* < 0.05), but positively correlated with daily average temperature. Daily average temperature was negatively correlated with PM_2.5_, SO_2_, NO_2_, CO, relative humidity, and air pressure (*r* < 0, *P* < 0.01) as shown in Table 2.

**Table 2:** The correlation between air pollutants and meteorological parameters.

**Variables**	**PM_2.5_**	**PM_10_**	**SO_2_**	**NO_2_**	**CO**	**O_3_-8h**	**Temperature**	**Humidity**	**Pressure**
PM_2.5_	1.000	0.773**	0.620**	0.608**	0.678**	−0.410**	−0.620**	0.179**	0.477**
PM_10_		1.000	0.650**	0.616**	0.386**	−0.190**	−0.339**	−0.131*	0.232**
SO_2_			1.000	0.608**	0.281**	−0.362**	−0.573**	−0.344**	0.531**
NO_2_				1.000	0.485**	−0.290**	−0.404**	0.005	0.372**
CO					1.000	−0.359**	−0.427**	0.525**	0.367**
O_3_-8h						1.000	0.841**	−0.191**	−0.749**
Temperature							1.000	−0.103*	−0.894**
Humidity								1.000	0.001
Pressure									1.000

** P < 0.01; *P < 0.05.

### Time series of air pollutants concentrations

**Figure 1:**
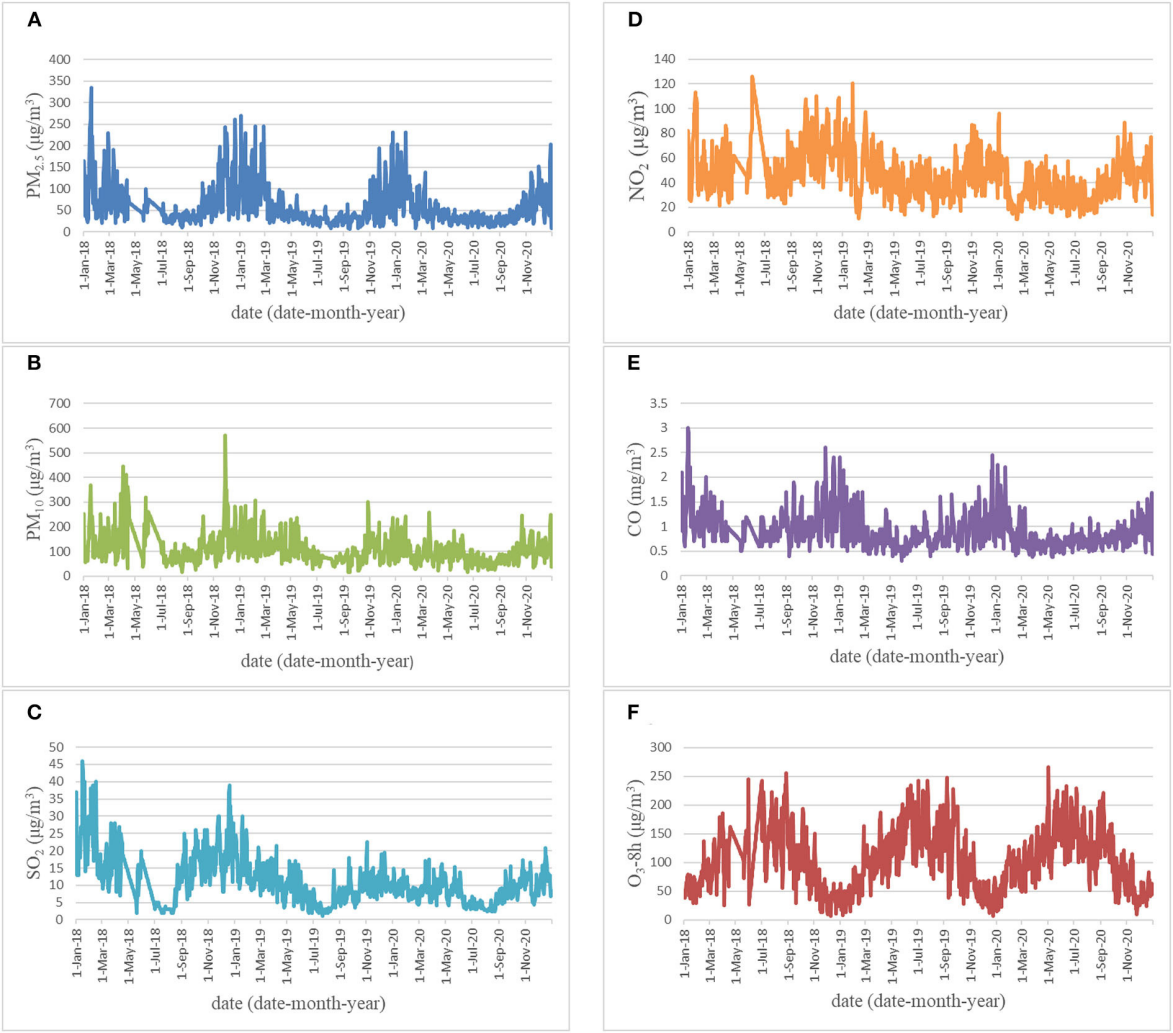
Time series of air pollutants [**(A)** PM_2.5_; **(B)** PM_10_; **(C)** SO_2_; **(D)** NO_2_; **(E)** CO; **(F)** O_3_-8h] concentrations in Zhengzhou, China.

Figure 1 displays the time series of air pollutants concentrations. PM_2.5_, PM_10_, SO_2_, NO_2_, and CO have similar cyclical fluctuation characteristics. The highest monthly average concentrations of PM_2.5_, PM_10_, SO_2_, NO_2_, and CO pollutants in the air were observed in January and February, the lowest concentrations were in July and August. The seasonal pattern showed low concentration in summer and high concentration in winter, but the concentration of O_3_-8h is high in summer and low in winter.

### Single-pollutant model

**Figure 2:**
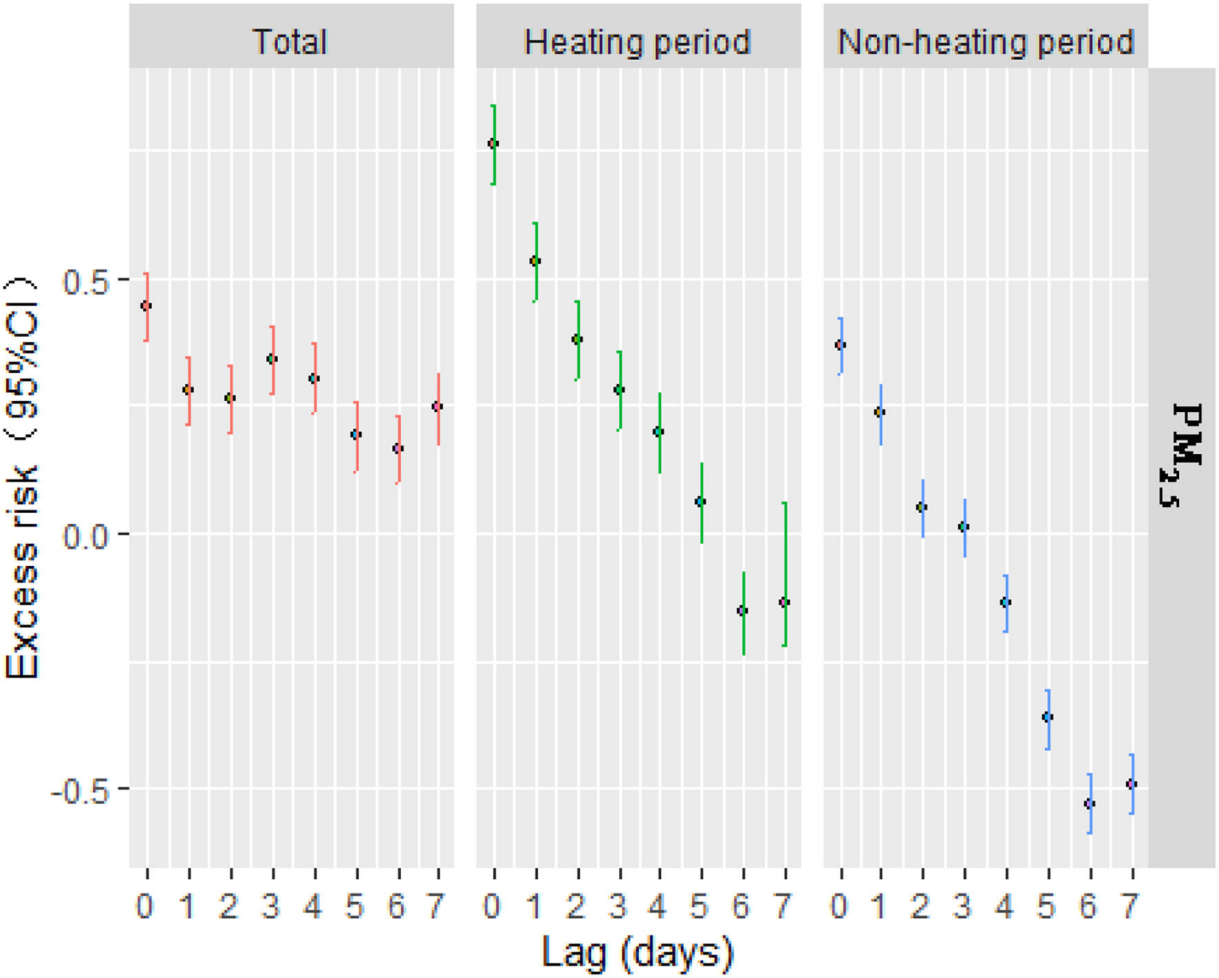
The lag effect of ambient PM_2.5_ and children's respiratory diseases in daily visits.

Table 3 indicated the increase in pollutant concentration on the same day significantly increased the risk of outpatient visits, and there was a certain lag effect. The impact of PM_2.5_ on the outpatient visits of children with respiratory diseases was statistically significant at lag1–7, and the effect was the strongest at lag3. For each increase in the concentration by 10 μg/m^3^, the risk of visits for children's respiratory diseases increased by 0.341% (95%*CI*: 0.274–0.407%) (Figure 2).

**Table 3:** Excess risk of ambient PM_2.5_ to outpatient visits of respiratory diseases in children (single pollution model, %).

**Lag days**	**Total population**		**Heating period**		**Non–heating period**	
	***ER***	**95%*CI***	***ER***	**95%*CI***	***ER***	**95%*CI***
0	0.443	0.377–0.509*	0.760	0.683–0.836*	0.366	0.310–0.421*
1	0.279	0.213–0.346*	0.532	0.455–0.609*	0.233	0.177–0.289*
2	0.262	0.195 −0.328*	0.380	0.303–0.457*	0.050	−0.006–0.106
3	0.341	0.274–0.407*	0.280	0.202–0.358*	0.012	−0.044–0.068
4	0.303	0.236–0.370*	0.198	0.119–0.276*	−0.137	−0.193–0.081
5	0.190	0.123–0.257*	0.060	−0.019–0.138	−0.364	−0.421–0.308
6	0.165	0.097–0.232*	−0.156	−0.235–.077*	−0.530	−0.587–0.473
7	0.245	0.177–0.313*	0.138	0.217–0.060*	0.493	0.550–0.436

* P < 0.05.

Figure 2 shows that the increase in pollutant concentration on the same day significantly increased the risk of outpatient visits during the heating period, and there was a certain hysteresis effect. The impact of PM_2.5_ on outpatient visits were statistically significant at lag1–4, lag6-7, and the effect was the strongest at lag1. For each 10 μg/m^3^ increase in PM_2.5_, the risk of children's respiratory disease visits increased by 0.532% (95% *CI*: 0.455%−0.609%) at lag1 but was not statistically significant at lag5.

The figure also shows that the increase in pollutant concentration on the same day significantly increase the risk of outpatient visits during the non-heating period, and there was a certain lag effect. The impact of PM_2.5_ on outpatient visits for respiratory diseases was statistically significant at lag1, the risk of children's respiratory disease visits increased by 0.233% (95% *CI*: 0.177–0.289%) for each 10 μg/m^3^ increase in the concentration, there was no statistical significance at lag2–7.

### Exposure response curve

**Figure 3:**
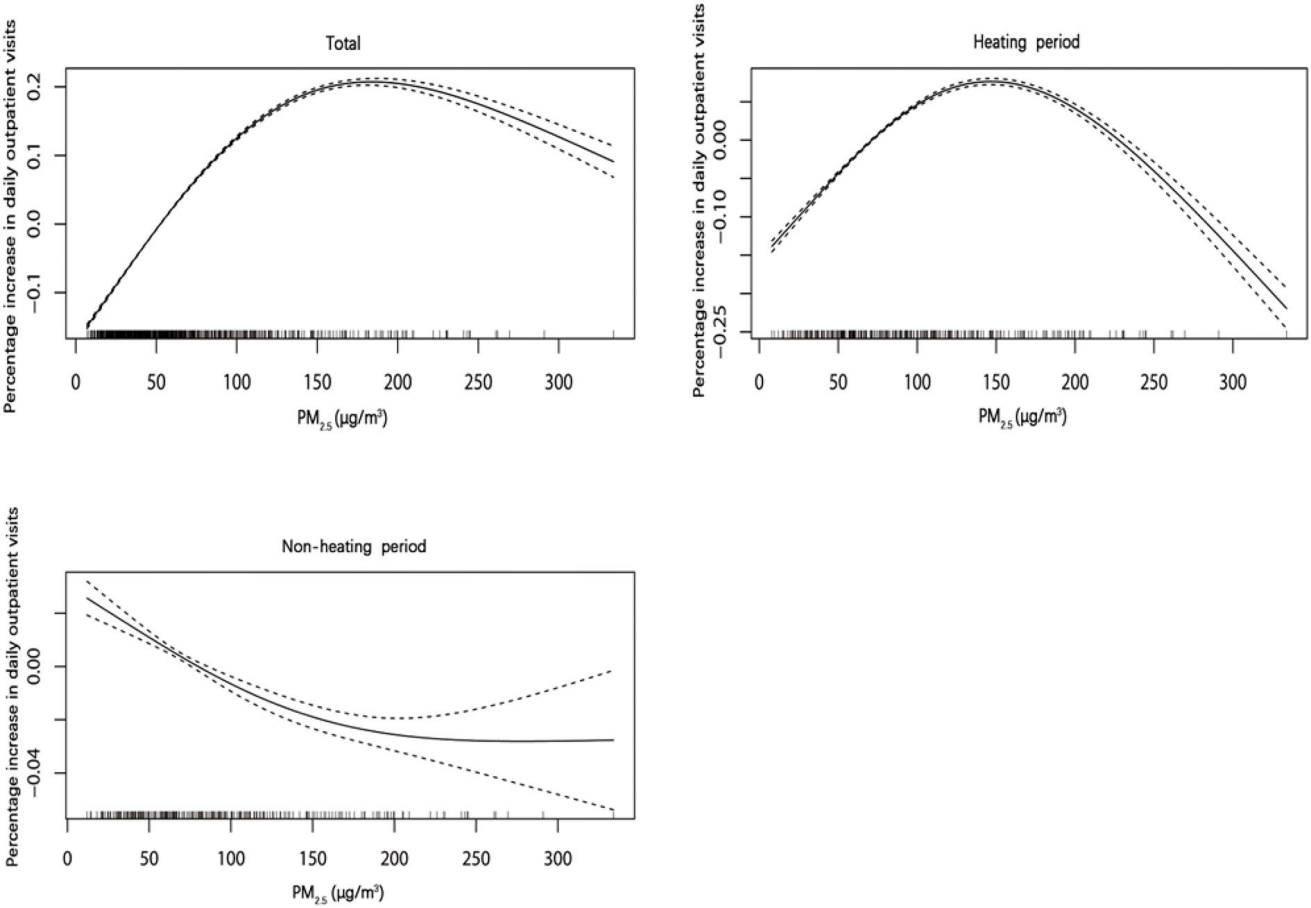
The relationship between exposure response of PM_2.5_ and outpatient visits for respiratory diseases in children.

The pollutant concentration on the maximum lag days was selected as the exposure level. Figure 3 shows the exposure response relationship between PM_2.5_ and children's respiratory diseases. The lag days of PM_2.5_ on the number of outpatient visits for children's respiratory diseases, outpatient visits during the heating period and the non-heating period were lag3, lag1, and lag1, respectively. We found that the exposure-response curves of the total population and the heating period show a non-linear slow rise. Under high concentration, the outpatient visit risk of PM_2.5_ to the total population tends to moderate after a short plateau period.

### Multi-pollutant model

In actual life, the impact of air pollutants on the health of residents is usually not caused by exposure to a single pollutant, but the result of the combined action of multiple pollutants. Therefore, when studying the impact of air pollution on the health of residents, it is necessary to consider using a multi-pollutant model for analysis to make the research results more reliable. As shown in Table 4, the data analysis of 3-day outpatient visits of children with respiratory diseases in the single pollutant model with the largest lag effect of PM_2.5_ showed that in the whole population, PM_2.5_ includes PM_10_ alone, or SO_2_, CO, O_3_-8h at the same time, the excess risk of outpatient visits for children with respiratory diseases caused by other pollutants was statistically significant (*P* < 0.05). When SO_2_ was controlled alone, the risk of PM_2.5_ on outpatient visits of children with respiratory diseases was consistent with a single pollutant model, that is, when PM_2.5_ increased by 10 μg/m^3^, the excess risk of children's respiratory disease visits increased by 0.220%(95%*CI*:0.147–0.294%) at lag3.

**Table 4:** Excess risk of ambient PM_2.5_ to outpatient visits of respiratory diseases in children (multipollution model, %).

**Pollutant**	**Total population**		**Heating period**		**Non-heating period**	
	***ER***	**95%*CI***	***ER***	**95%*CI***	***ER***	**95%*CI***
**Double pollutants**						
+SO_2_	0.220	0.147–0.294*	0.737	0.650–0.823*	0.229	0.533–0.076
+CO	0.399	0.494–0.303*	0.473	0.345–0.600*	0.049	0.367–0.269
+PM_10_	0.081	0.180–0.018	0.065	0.186–0.056	1.998	2.371–1.624
+NO_2_	0.225	0.301–0.149*	0.142	0.053–0.231 *	0.378	0.074–0.683*
+O_3_8h	0.409	0.343 −0.475*	0.726	0.649–0.803 *	0.921	0.627–1.216 *
**Three pollutants**						
+SO_2_+CO	0.451	0.548–0.354*	0.475	0.346 −0.603*	0.596	0.920–0.271*
+SO_2_+PM_10_	0.226	0.328–0.124*	0.089	0.217 −0.038	3.016	3.403–2.629
+SO_2_+NO_2_	0.202	0.280–0.124*	0.276	0.183–0.368 *	0.253	0.565–0.060
+SO_2_+O_3_8h	0.196	0.122 −0.269*	0.695	0.608–0.782*	0.307	0.623–0.010
+CO+PM_10_	0.966	1.088–0.844*	0.443	0.605–0.280*	3.109	3.511–2.707*
+CO+NO_2_	0.738	0.836–0.640*	0.009	0.141–0.124	0.355	0.684–0.026*
+CO+O_3_8h	0.448	0.543–0.352*	0.528	0.400–0.657 *	0.096	0.425–0.235
+PM_10_+NO_2_	0.484	0.586–0.381*	0.582	0.710–0.455*	2.153	2.53–1.774 *
+PM_10_+O_3_8h	0.172	0.272–0.072*	0.053	0.174–0.068	2.080	2.464–1.695*
**Four pollutants**						
+SO_2_+CO+PM_10_	0.980	1.102–0.858*	0.439	0.602–0.276*	3.491	3.895–3.085*
+SO_2_+CO+NO_2_	0.721	0.819–0.623*	0.004	0.136–0.128	0.605	0.935–0.274
+SO_2_+CO+O_3_ 8h	0.498	0.595–0.401	0.524	0.395–0.653	0.668	1.004–0.332*
+CO+PM_10_+NO_2_	1.065	1.188–0.943*	0.812	0.977–0.647*	3.129	3.531–2.726*
+CO+PM_10_+O_3_ 8h	1.089	1.212–0.967*	0.370	0.535–0.205*	3.197	3.608–2.784*
+PM_10_+NO_2_+O_3_ 8h	0.596	0.699–0.493*	0.570	0.697–0.442*	2.251	2.640–1.860*
+NO_2_+O_3_ 8h+SO_2_	0.249	0.327–0.171*	0.271	0.179–0.363*	0.336	0.661–0.010*
**Five pollutants**						
+SO_2_+CO+PM_10_+NO_2_	1.065	1.188–0.943*	0.812	0.977–0.647*	3.129	3.531–2.726*
+SO_2_+CO+PM_10_+O_3_ 8h	1.089	1.212–0.967*	0.370	0.535–0.205*	3.197	3.608–2.784*
+CO+PM_10_+O_3_ 8h+NO_2_	1.203	1.326–1.080*	0.785	0.953–0.617*	0.061	0.378–0.502
**All the pollutants**						
PM_2.5_+SO_2_+CO+ PM_10_+NO_2_+O_3_ 8h	1.198	1.321–1.075*	0.812	0.980–0.643*	0.061	0.380–0.503

* P < 0.05.

During the heating period, PM_2.5_ was added into PM_10_ alone, or both SO_2_ and PM_10_, or with SO_2_, CO, and O_3_-8h, except for SO_2_, CO, and NO_2_, the excess risk of outpatient visits for children with respiratory diseases caused by other pollutants was statistically significant. When SO_2_ was controlled alone, the excess risk of daily outpatient visits of children with respiratory diseases increased by 0.737% (95%*CI*: 0.650–0.832%).

During the non-heating period, the analysis found that in addition to NO_2_ or all pollutants at the same time, the total outpatient risk of children's respiratory diseases caused by other pollutants was statistically significant, and when O_3_-8h was individual controlled, PM_2.5_ will increase the risk of daily outpatient visits for children with respiratory diseases by 0.921% (95%*CI*: 0.627–1.216%) (Table 4).

## Discussion

Air pollution is directly or indirectly related to many health effects. Children are in the period of growth and lung function development. They are vulnerable to adverse factors of the external environment. They have become one of the most sensitive groups of air pollution (22, 23). Although the link between air pollution and outpatient visits for respiratory diseases in children has been extensively studied in the developed countries, the studies are limited in developing countries, especially in the cities with heavily polluted air. Air particulate matter, especially the relationship between air particulate matter PM_2.5_ and respiratory diseases has become a hot research topic. Our results showed that the increase of PM_2.5_ would increase the number of visits for respiratory diseases in children, and the impact was more sensitive during the heating period than during the non-heating period.

Our study found that the concentration of PM_2.5_, PM_10_, and NO_2_ in Zhengzhou exceeded the second-level concentration limit of the AQS. The possible reason is that the number of motor vehicles in Zhengzhou has increased sharply in recent years, and automobile exhaust emissions have increased. Correlation analysis between meteorological factors and atmospheric pollutant concentration shows that PM_2.5_, PM_10_, SO_2_, NO_2_, and CO pollutants are negatively correlated with daily average temperature, which further clarifies the reason why pollution is more serious in winter in Zhengzhou. The reason may be that Zhengzhou provides heating in winter; therefore, a large amount of coal is burned. Additionally, it is easy to form temperature inversion in the winter because the surface temperature is low, which makes it less likely to diffuse pollutants (24).

Domestic and foreign studies have shown that PM_2.5_ exposure is related to the number of respiratory disease visits, but the research results of PM_2.5_ on the increase of children's respiratory disease visits are not consistent. The levels of air pollutants observed in our study is far higher than the previous health research in developed countries (22, 23). The single-day lag model of PM_2.5_ on the total population shows that PM_2.5_ has the strongest effect at a 3-day lag. Every 10μg/m^3^ increase in PM_2.5_ is associated with a 0.341% (95%*CI*: 0.274–0.407%) increased in the outpatient visits of children with respiratory diseases. The impact of PM_2.5_ in our study is slightly different from the results in other cities or regions (25–27). This may be related to the different geographical location and regional characteristics. Therefore, it is suggested that when we compare the impacts of PM_2.5_ from different studies, the regional distribution and seasonal factors should be comprehensively considered. However, regardless of the degree of impact, the results of this study and other research (28, 29) shows that PM_2.5_ exposure may increase the number of outpatient children with respiratory diseases. The higher the concentration of pollutants, the stronger the damage to the body. Therefore, children should reduce outdoor activities during air pollution, especially during haze, to avoid or reduce the adverse effects of PM_2.5_ exposure on health. The exposure response relationship between PM_2.5_ and the number of children with respiratory diseases showed a non-linear exposure response curve. This indicates a higher concentration of PM_2.5_ may lead to a significant increase in the number of visits for respiratory diseases in children, even at low environmental concentrations.

The analysis of the multi-pollutant model is mainly used to study the difference in the influence of different pollutant combinations on the increase of outpatient visits of children with respiratory diseases. Most previous studies introduced only three or two pollutants in PM_2.5_, PM_10_, SO_2_ and NO_2_ into the multi-pollutant model (30), but in this study, six common pollutants PM_2.5_, PM_10_, SO_2_, NO_2_, CO, and O_3_−8h in atmospheric air quality monitoring were all introduced into the model for analysis, but the effect was lower than that of the single pollutant model. After including SO_2_, CO, O_3_-8h or SO_2_, CO, NO_2_ in the model, PM_2.5_ effects attenuated to null, suggesting that PM_2.5_ may not be an independent risk factor to children's respiratory health. There may be two reasons: First, because of the complicated correlation between air pollutants, when one pollutant is the research object, the other pollutants are all confounding factors. In a multivariate linear model, the possible collinearity between pollutants affects the estimated value of the effect (31). Second, whether gaseous pollutants (CO, SO_2_, NO_2_) should be included in the multi-pollutant model as confounding factors of the health effects of particulate matter needs further studies.

Our study showed that after adjusting for potential confounding factors, compared with the non-heating period, the negative health effects of ambient PM_2.5_ on the outpatient visits for children with respiratory diseases increased during the heating period. Although the exact mechanism is not well understood, the association between PM_2.5_ and outpatient visits of children with respiratory diseases is biologically plausible. On one hand, children's alveolar epithelium is not fully developed, easy to cause, has high permeability, and breathes 50% more air per kilogram of body weight than adults, which are susceptible (32). On the other hand, PM_2.5_ has been demonstrated to produce reactive oxygen species that can trigger the inflammatory processes in the respiratory tract (33). Furthermore, a large amount of coal is used for heating and the atmospheric boundary layer is low in winter. During the combustion process, coal emits a large amount of sulfur dioxide and dust particles are emitted. Thus, it is difficult for air pollutants to be diffused and transported. In recent years, with rapid development of socioeconomics and urbanization, there are a growing number of motor vehicles in the urban areas, which elevates the air pollutant concentration due to the traffic exhaust. In addition, unfavorable meteorological conditions, such as low rainfall and relatively stable weather, would lead to the accumulation of air pollutants.

There are some limitations in our study. First, we only collected data for three years to examine the impact of PM_2.5_ on the outpatient visits of children with respiratory diseases. Second, we used the air pollution data of nine national environmental monitoring stations to reflect the situation of children's exposure to air pollutants, which may misestimate the individual actual exposures. Measurement errors may have a significant impact on the interpretation of epidemiological studies of air pollution (34). However, due to the difference in the sources and levels of air pollution, our findings in this study should be interpreted carefully for other megacities in the world.

## Conclusions

We found that the level of PM_2.5_ significantly increased the risk of children's respiratory diseases. The results indicate that particulate matter may cause damage to children's respiratory system. Therefore, parents should pay attention to children's protective measures and try to reduce outdoor activities when the concentration of air pollutants increases. Meanwhile, the government should strengthen environmental protection and popularize the “coal to gas” project, so as to reduce the emission of pollutants and promote public health, especially the health of children.

## Data availability statement

The raw data supporting the conclusions of this article will be made available by the authors, without undue reservation.

## Ethics statement

The studies involving human participants were reviewed and approved by Ethics Review Committee of Zhengzhou University (2020–47). The patients/participants provided their written informed consent to participate in this study.

## Author contributions

LL and YH conceived the idea of the study. LL, BW, NQ, HW, LW, and GY collected and analyzed the data. LL drafted the manuscript and approved the final manuscript. YH revised and improved the manuscript. All authors have read and agreed to the published version of the manuscript.

## Funding

This study was supported by the 2021 Henan Province Key R&D and Promotion Special Project (No. 212102310814), National Key R&D Program for Active Health and Aging Science and Technology (No. 2020YFC2006100), and Annual Project of Philosophy and Social Sciences of Henan Province (2020BSH017).

## Conflict of interest

The authors declare that the research was conducted in the absence of any commercial or financial relationships that could be construed as a potential conflict of interest.

## Publisher's note

All claims expressed in this article are solely those of the authors and do not necessarily represent those of their affiliated organizations, or those of the publisher, the editors and the reviewers. Any product that may be evaluated in this article, or claim that may be made by its manufacturer, is not guaranteed or endorsed by the publisher.
